# Leucine modulation of mitochondrial mass and oxygen consumption in skeletal muscle cells and adipocytes

**DOI:** 10.1186/1743-7075-6-26

**Published:** 2009-06-05

**Authors:** Xiaocun Sun, Michael B Zemel

**Affiliations:** 1 University of Tennessee, Knoxville, Tennessee, 37996, USA

## Abstract

**Background:**

The effects of dairy on energy metabolism appear to be mediated, in part, by leucine and calcium which regulate both adipocyte and skeletal muscle energy metabolism. We recently demonstrated that leucine and calcitriol regulate fatty acid oxidation in skeletal muscle cells *in vitro*, with leucine promoting and calcitriol suppressing fatty acid oxidation. Moreover, leucine coordinately regulated adipocyte lipid metabolism to promote flux of lipid to skeletal muscle and regulate metabolic flexibility. We have now investigated the role of mitochondrial biogenesis in mediating these effects.

**Methods:**

We tested the effect of leucine, calcitriol and calcium in regulation of mitochondrial mass using a fluorescence method and tested mitochondrial biogenesis regulatory genes as well mitochondrial component genes using real-time PCR. We also evaluated the effect of leucine on oxygen consumption with a modified perfusion system.

**Results:**

Leucine (0.5 mM) increased mitochondrial mass by 30% and 53% in C2C12 myocytes and 3T3-L1 adipocytes, respectively, while calcitriol (10 nM) decreased mitochondrial abundance by 37% and 27% (p < 0.02). Leucine also stimulated mitochondrial biogenesis genes SIRT-1, PGC-1α and NRF-1 as well as mitochondrial component genes UCP3, COX, and NADH expression by 3–5 fold in C2C12 cells (p < 0.003). Adipocyte-conditioned medium reduced mitochondrial abundance (p < 0.001) and decreased UCP3 but increased PGC-1α expression in myocytes, suggesting a feedback stimulation of mitochondrial biogenesis. Similar data were observed in C2C12 myocytes co-cultured with adipocytes, with co-culture markedly suppressing mitochondrial abundance (p < 0.02). Leucine stimulated oxygen consumption in both C2C12 cells and adipocytes compared with either control or valine-treated cells. Transfection of C2C12 myocytes with SIRT-1 siRNA resulted in parallel suppression of SIRT-1 expression and leucine-induced stimulation of PGC-1α and NRF-1, indicating that SIRT-1 mediates leucine induced mitochondrial biogenesis in muscle cells.

**Conclusion:**

These data suggest that leucine and calcitriol modulation of muscle and adipocyte energy metabolism is mediated, in part, by mitochondrial biogenesis.

## Background

Previous studies demonstrate that dairy foods may inhibit adiposity [1,2]; this effect is mediated, in part, by dietary calcium suppression of calcitriol (1,25-(OH)_2_-D_3_) which otherwise promotes lipogenesis and inhibits lipolysis via both genomic and non-genomic mechanisms [3-5]. Indeed, vitamin D receptor (VDR) knockout mice exhibited a lean phenotype and resistance to diet-induced obesity [6]. Dairy foods also contain significant non-calcium anti-obesity bioactivity [1,2]; this is largely attributable to leucine, which we have recently found to exert significant effects on both adipocyte and skeletal muscle energy metabolism [7]. Notably, dietary calcium and dairy induced lipolysis is not associated with hyperlipidemia [8], suggesting a coupling with fatty acid oxidation. These observations are consistent with our recent data which indicate that dietary calcium and dairy reduce inflammatory and oxidative stress [9,10], which otherwise are commonly found in hyperlipidemic conditions [11].

Skeletal muscle constitutes an important site for lipid utilization, and we have recently demonstrated that leucine and calcitriol participate in the regulation of fatty acid oxidation in skeletal muscle cells *in vitro*, with leucine promoting fatty acid oxidation while calcitriol exerts the opposite effect [7]. In addition, leucine also modulated adipocyte lipid metabolism, possibly serving to provide an increased flux of lipid to skeletal muscle, thereby providing the energy substrate to support leucine-stimulated protein synthesis. However, the mechanism underlying the effects of leucine and calcitriol on skeletal muscle fatty acid oxidation is not clear. Notably, skeletal muscle fatty acid oxidation appears to be associated with mitochondrial biogenesis and expression of multiple genes, such as peroxisome proliferator-activated receptor gamma coactivator 1-alpha (PGC-1α) and sirtuins, which are involved in the regulation of energy metabolism via their modulation of thermogenesis, mitochondrial number and fatty acid oxidation [12-14].

Accordingly, this project was designed to investigate the role of leucine and calcitriol in regulation of mitochondrial biogenesis and expression of genes involved in modulation of mitochondrial biogenesis and energy metabolism in skeletal muscle cells and adipocytes. To further test the physiological consequences related to mitochondrial biogenesis and energy metabolism, we also assessed the effects of leucine on cellular oxygen consumption in both cell types.

## Materials and methods

### Experimental Approach

We first measured mitochondrial mass using NAO fluorescent dye in differentiated muscle cells and adipocytes to explore the direct effect of leucine and calcitriol in regulation of mitochondrial mass. We also evaluated the expression of well-recognized regulatory genes in mitochondrial biogenesis such as SIRT-1, NRF and PGC-1α, as well as mitochondrial component genes such as NADH dehydrogenase, cytochrome C oxidase, and UCP3 in muscle cells alone, or in muscle cells pretreated with conditioned medium previously collected from adipocyte culture or co-cultured with muscle cells. Conditioned medium treatment and co-culture have been demonstrated to be an effective tool to investigate the cross-talk via secretory factors in metabolism between two different cell types. To further evaluate the effect of leucine in regulating mitochondrial function, we also designed a novel oxygen consumption system that measured oxygen consumption in both muscle cells and adipocytes. Since SIRT-1 is a key regulatory gene on mitochondrial biogenesis, we also did a SIRT-1 knock-down study using siRNA to determine whether SIRT-1 mediates the effect of leucine and calcitriol on mitochondrial mass. In some experiments, the calcium channel antagonist nifedipine was used to investigate the role of calcium signaling in regulation of calcitriol modulation of mitochondrial mass. The concentration of leucine selected for these studies (0.5 mM), as well as our previous work [7], is based upon typical plasma levels achieved following a high protein meal or following leucine administration in mice (0.4 – 0.6 mM) [15-17]. To address the possibility that the effects of leucine are non-specific effects of branched chain amino acids as an energy substrate, key experiments incorporated the same concentration of valine as an additional control.

### Cell culture

C2C12 and 3T3-L1 preadipocytes (American Type Culture Collection) were plated at a density of 8000 cells/cm^2 ^(10 cm^2 ^dish) and grown in Dulbecco's modified eagle's medium (DMEM) containing 10% fetal bovine serum (FBS), and antibiotics (growth medium) at 37°C in 5% CO_2_. Confluent 3T3-L1 preadipocytes were induced to differentiate with a standard differentiation medium consisting of DMEM medium supplemented with 10% FBS, 250 nM dexamethasone, 0.5 mM 3-Isobutyl-1-methylxanthine (IBMX) and 1% penicillin-streptomycin. Preadipocytes were maintained in this differentiation medium for 3 days and subsequently cultured in growth medium. Cultures were re-fed every 2–3 days to allow 90% cells to reach fully differentiation before conducting chemical treatment. For differentiation of C2C12 cells, cells were grown to 100% confluence, changed into differentiation medium (DMEM with 2% horse serum and 1% penicillin-streptomycin), and fed with fresh differentiation medium every day until myotubes were fully formed (3 days). In some experiments, cells were seeded on 40 mm coverslips (Bioptechs Inc., Butler, PA) which were loaded later in the FSC2 incubator as described in the oxygen consumption measurement section.

### Co-culture of adipocyte and C2C12

Cells were co-cultured by using transwell inserts with a 0.4 μm porous membrane (Corning, Lowell, MA) to separate adipocytes and C2C12 muscle cells as described previously [10]. After incubation for 48 hours, the cells in the lower well were harvested for further analysis.

### Treatment of cells

Calcitriol, leucine, valine and nifedipine were freshly diluted in medium before treatment. Cells were incubated in serum free medium overnight and then washed with fresh medium, re-fed with medium containing the different treatments (0.5 mM leucine, 0.5 mM valine and/or 5 μM nifedipine with or without 10 nM calcitriol) and incubated at 37°C in 5% CO_2 _for 48 h before analysis. In some experiments, the supernatants of differentiated 3T3-L1 adipocytes (conditioned medium) were used to replace the medium of C2C12 myocytes. Cell viability was measured via trypan blue exclusion.

### Transfection

siRNA-annealed oligonucleotide duplexes for SIRT1 (Sequence 5'->3' sense: GCAAUAGGCCUCUUAAUUAtt; antisense: UAAUUAAGGCCUAUUGCtt) and negative control (Catalog NO. 4611) were purchased from Ambion (Ambion, Austin, Texas, USA) and C2C12 cells were transfected using siPORT NeoFX (Ambion, Austin, TX) following the manufacturer's instructions.

### Total RNA extraction

A total cellular RNA isolation kit (Ambion, Austin, Texas, USA)) was used to extract total RNA from cells according to manufacturer's instruction. The concentration and purity of the isolated RNA were measured spectrophotometrically (A_280_/A_260 _between 1.9–2.1) and the integrity of RNA sample were analyzed via BioAnalyzer (Agilent 2100, Agilent Technologies).

### Quantitative real-time PCR

Adipocyte and muscle 18S, genes encoding mitochondrial component protein such as cytochrome c oxidase (COX) subunit VIIc1, NADH dehydrogenase (NADH) and uncoupling protein 3 (UCP3), and mitochondrial biogenesis regulatory genes PGC-1α, nuclear respiratory factor-1 (NRF), and sirtuin 1 (SIRT-1) were quantitatively measured using an ABI 7300 Real-Time PCR System (Applied Biosystems, Branchburg, New Jersey, USA) with a TaqMan 1000 Core Reagent Kit (Applied Biosystems, Branchburg, New Jersey, USA). The primers and probe sets were obtained from Applied Biosystems TaqMan^® ^Assays-on-Demand™ gene expression primers and probe sets according to manufacture's instruction. Pooled adipocyte total RNA were serial-diluted in the range of 1.5625–25 ng and used to establish a standard curve; total RNAs for unknown samples were also diluted in this range. Reactions of quantitative RT-PCR for standards and unknown samples were performed according to the instructions of ABI 7300 Real-Time PCR System and TaqMan Real Time PCR Core Kit. The mRNA quantitation for each sample was further normalized using the corresponding 18S quantitation.

### Mitochondrial mass assay

The mitochondrial probe NAO (Invitrogen, Carlsbad, California, USA) was used to analyze mitochondrial mass by fluorescence (excitation 485 nm and emission 520 nm). Qualitative imaging data were obtained using a fluorescence microscope (Leica, Lasertechnik GmbH, Heidelberg, Germany) linked to a Hamamatsu color chilled 3CCD camera (Hamamatsu, Japan), and quantitative data were obtaining with a fluorescence microplate reader (Packard Instrument, Downers Grove, Illinois, USA). The intensity of fluorescence was expressed as arbitary units per μg protein.

### Oxygen consumption measurement

**Figure 1:**
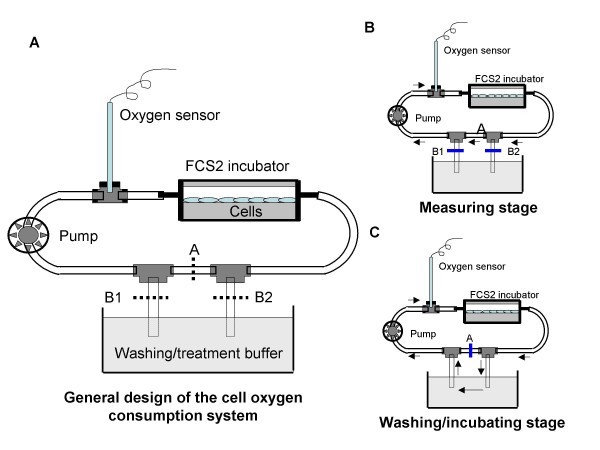
**Schematic figures of the oxygen consumption system**. A) The general design of the oxygen consumption system; B) measurement stage; C) washing stage.

To monitor the real-time oxygen consumption by C2C12 and adipocyte, we designed an *in vitro *oxygen consumption system as shown in figure 1A. Coverslips containing either adherent C2C12 cells or differentiated adipocytes, which had been pre-treated with reagents as described, were loaded into an FCS2 stage incubator (Bioptechs Inc., Butler, PA) The FCS2 incubator is a closed chamber with a perfusion pathway formed by separating the microaqueduct slide from the coverslip containing cells with a single silicone gasket to generate laminar flow conditions during perfusion. The FCS2 incubator was connected to a peristaltic pump on the efferent side using 1/16" C-Flex Tubing (Bioptechs Inc., Butler, PA). A dissolved oxygen meter (Warner Instruments, Hamden, CT) was inserted with a polypropylene T-shaped connector and sealed in the perfusion pathway to detect the changes in oxygen concentration in the perfusion fluid (RPMI 1640). By controlling of the three clamps located on the tubing, the perfusion pathway can be set as sealed circulation for either oxygen consumption measurement (Figure 1B) or washing. The sealed circulation design blocks oxygen coming from outside the pathway and provides continuous mixing to ensure uniform oxygen concentrations during measurement. The oxygen sensor was pre-calibrated in gas and aqueous phases prior to each experiment. The total fluid in the closed circulation was 2 ml. In contrast to previous studies investigating cellular oxygen consumption [18-20], our system presents a novel approach in it's ability to continuously measure real-time oxygen consumption in adherent cells with small amount of fluid (<2 ml) in a sealed circulation using relatively inexpensive equipment.

The oxygen sensor measures the monotonic decrease in dissolved oxygen in parts per million (ppm) over time due to oxygen consumption and the data were fit to curves using polynomial regression, and initial rates of O_2 _consumption were calculated from the first three minutes of each test. Cell counts were done via hemocytometer to assure that equivalent cell numbers were utilized for all treatments (~1 × 10^6 ^cells).

### Statistical analysis

All data are presented as mean ± SD. Data were evaluated by one-way or two-way ANOVA as appropriate for each experiment and linear regression test for the first 3 min of oxygen consumption data, and significantly different group means (p < 0.05) were then separated by the least significant difference test using SPSS (SPSS Inc, Chicago, IL).

## Results

**Figure 2:**
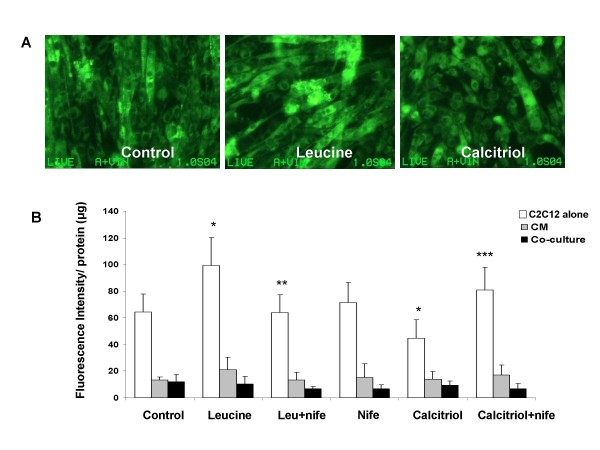
**The effects of leucine, calcitriol and nifedipine on mitochondrial mass as assessed by NAO dye-binding as described in material and methods section in C2C12 myocytes treated with or without conditioned medium (CM) derived from differentiated adipocytes or co-cultured with differentiated 3T3-L1 adipocytes**. The upper panel (A) shows fluorescent images of mitochondrial density and the lower panel shows the quantitative data in response to the treatments. Values are presented as mean ± SD, n = 6. Means with * differ with control(p < 0.005), ** differ with leucine (p = 0.01), *** differ with calcitriol (p < 0.001).

**Figure 3:**
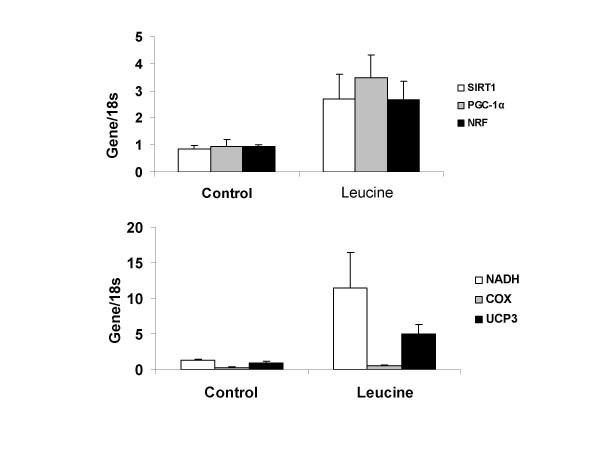
**The effect of leucine on SIRT-1, PGC-1α, NRF, NADH, COX, and UCP3 gene expression in C2C12 myocytes; expression of each gene is normalized to 18s expression**. Cells were treated with or without leucine (0.5 mM) for 48 hours. Values are presented as mean ± SD, n = 6. Means with * differ compared with control with p < 0.005.

**Figure 4:**
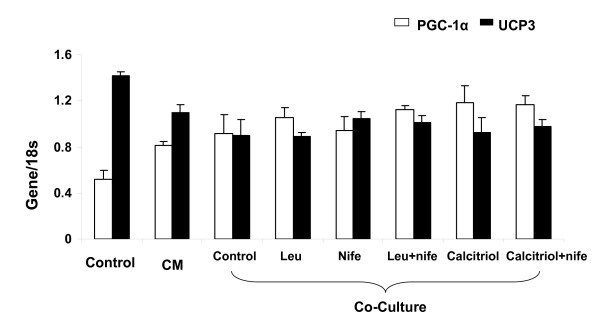
**The effect of leucine, and/or nifedipine and/or calcitriol on PGC-1α and UCP3 gene expression in C2C12 myocytes pretreated with CM derived from adipocytes or co-cultured with differentiated with 3T3-L1 adipocytes**. Cells were treated with or without leucine, nifedipine, or/and calcitriol for 48 hours. Values are presented as mean ± SD, n = 6. Means with * differ compared with control PGC-1α and ** differ compared with control UCP3 with p < 0.001.

**Figure 5:**
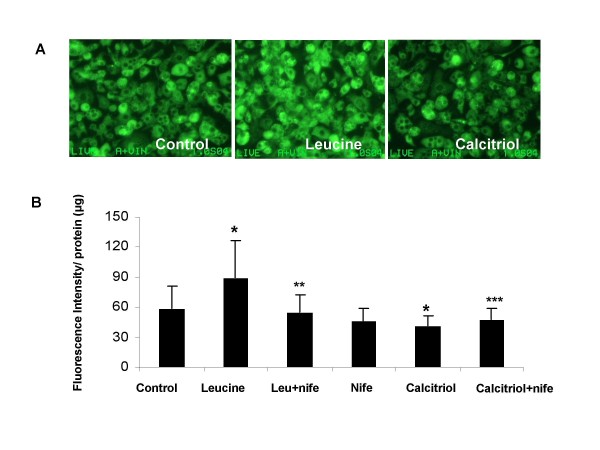
**The effect of leucine, and/or nifedipine and/or calcitriol on mass by NAO binding in differentiated 3T3-L1 adipocytes**. The upper panel (A) shows fluorescent images of mitochondrial density and the lower panel (B) shows the quantity data in response to the treatments. Cells were treated with or without leucine (0.5 mM), nifedipine (10 μM), or/and calcitriol (10 nM) for 48 hours. Values are presented as mean ± SD, n = 6. Means with * differ compared with control group with p < 0.04. ** differ with leucine (p < 0.05), *** differ with calcitriol (p < 0.05)

Leucine increased mitochondrial mass in C2C12 myocytes, while calcitriol exerted the opposite effect (Figure 2). Exposure of myocytes to adipocyte factors via either conditioned medium or co-culture with differentiated adipocytes markedly attenuated the effect of leucine on mitochondrial biogenesis in myocytes (Figure 1B, p < 0.001). Leucine treatment increased the expression of mitochondrial biogenesis regulatory genes SIRT-1, PGC-1α, NRF (Figure 3A, P < 0.05), and mitochondrial component genes NADH, COX and UCP3 (Figure 3B; p < 0.05), Further, adipocyte-conditioned medium and co-culture decreased UCP3 expression in myocytes while PGC-1α showed the opposite response (Figure 4; p < 0.001), suggesting a possible feedback up-regulation of mitochondrial biogenesis, although no effect was found in NRF or SIRT1(data not shown). Similar effects were found in differentiated 3T3-L1 adipocytes, as leucine increased mitochondrial mass while calcitriol exerted the opposite effect (Figure 5; P < 0.04); addition of the calcium channel antagonist nifedipine partially inhibited the effect of calcitriol in both cell types (Figure 2B and Figure 5B, p < 0.04).

**Figure 6:**
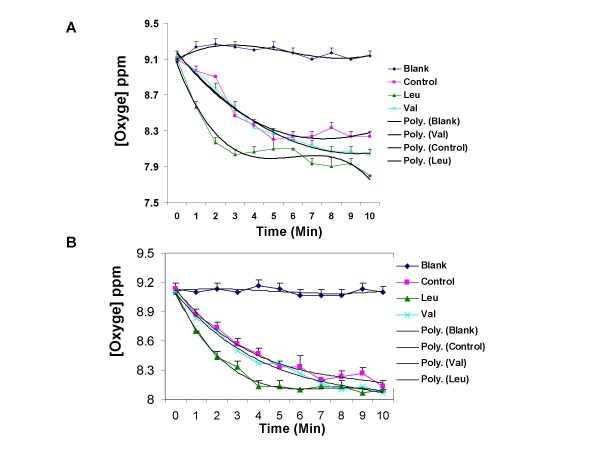
**The effect of leucine on oxygen consumption in (A) C2C12 cells and (B) 3T3-L1 adipocytes**. Cells were pre-treated with or without 0.5 mM leucine and data were presented as the dissolved oxygen concentration in perfusion fluid RPMI 1640. The "blank" depicts O_2 _in the absence of cells on the coverslip. The figures depict polynomial trendlines superimposed upon the data points. Initial rates were compared using data from the first 3 mins data of oxygen consumption in both cells types.

To further test the physiological significance of the observed regulation of mitochondrial biogenesis, we measured the oxygen consumption in C2C12 cells and differentiated adipocytes using our oxygen consumption system shown in Figure 5. Leucine significantly stimulated oxygen consumption in both C2C12 cells (Figure 6A) and adipocytes (Figure 6B) with leucine treatment resulting in an 89% increase in C2C12 and a 27% increase in adipocytes in the first 3 mins, respectively (p < 0.001). To verify the specificity of the leucine effect, we also assessed the effects of another branched chain amino acid (valine), and found it to exert no effect on oxygen consumption in either myocytes or adipocytes.

**Figure 7:**
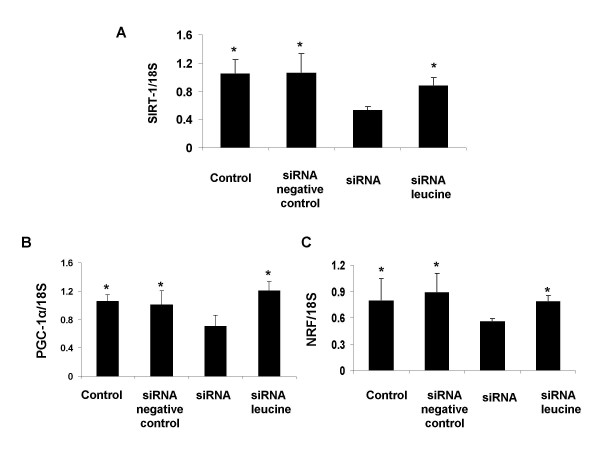
**The effect of leucine on SIRT-1 (A) PGC-1α (B) and NRF (C) expression in C2C12 myocytes with or without SIRT-1 siRNA transfection; expression of each gene is normalized to 18s expression**. Values are presented as mean + SD, n = 6. Means with * differ compared from siRNA groups at p < 0.05.

To further investigate the role of the regulatory genes in modulating leucine-induced mitochondrial biogenesis in myocytes, we knocked down SIRT-1 in C2C12 myocytes using siRNA. SIRT-1 siRNA transfection successfully decreased SIRT-1 mRNA by ~70% and correspondingly attenuated leucine induced SIRT-1 expression (Figure 7A, p < 0.05). Consistent with this, SIRT-1 knock-down reduced PGC-1α gene expression and attenuated leucine-induced PGC-1α gene expression (Figure 7B, p < 0.05), reduced NRF expression and abolished leucine-stimulation of NRF expression (Figure 7C, p < 0.05) in myocytes.

## Discussion

Data from the present study demonstrate that leucine increases mitochondrial mass and associated regulatory gene expression in both myocytes and adipocytes, while calcitriol exerts the opposite effect. Leucine also stimulated oxygen consumption in both myocytes and adipocytes, providing further evidence to support the role of leucine in regulation of energy combustion. However, exposure of myocytes to adipocytes via either conditioned medium or co-culture attenuates these effects, suggesting that one or more molecules produced by excess adipose tissue may play a role in suppressing skeletal muscle fatty acid oxidation by suppressing mitochondrial biogenesis.

Mitochondria play a key role in modulating adipocyte lipid metabolism and adipogenesis [22,23]. Metabolic disorders are associated with mitochondrial loss and dysfunction [24,25] and pharmacological strategies to induce mitochondrial proliferation improve insulin signaling and energy metabolism [26]. Key proteins of the mitochondrial respiratory chain are strongly reduced in adipose tissue, and reduced expression of oxidative phosphorylation genes and regulatory genes such as PGC-1α have been reported in diabetes [27]. However, the molecular signaling leading to this cellular energy metabolism dysfunction is largely unknown, although possible mechanisms have recently been proposed [26,28,29]. In addition, increased mitochondrial abundance induced by over-expression of NRF in adipose tissue increased synthesis of adiponectin [29], which has been shown to stimulate fatty acid combustion [30], while impaired mitochondrial function increased ER stress and reduced adiponectin transcription via activation of N-terminal kinase (JNK) [29].

PGC-1α is key nuclear receptor co-activator for mitochondrial biogenesis, and over-expression of this gene in mouse skeletal muscle increases mitochondrial abundance, especially in type II fiber rich muscles, resulting in increased energy expenditure and reduced body weight [31-33]. Further, recent data suggest that PGC-1α over-expression in rodents enables muscle to utilize lipid more efficiently [34]. Moreover, chronic physical activity has been demonstrated to increase mitochondrial biogenesis and oxidative muscle fiber content, and this effect is partially attributed to expression of PGC-1α [35]. Our data indicate that leucine increases mitochondrial biogenesis and PGC-1α expression, while calcitriol has the opposite effect, suggesting that leucine and calcitriol regulate skeletal muscle energy metabolism, in part, by modulating PGC-1α expression. Unlike PGC-1α, sirtuins are NAD+-dependent deacetylases that remove acetyl groups from acetyllysine-modified proteins, thereby regulating the biological function of their targets [36]. In mammals, SIRT-1 deacetylates histone proteins as well as non-histone proteins, and appears to function as an energy sensor linking energy metabolism to transcriptional regulation [37]. SIRT-1 regulates PGC-1α and mitochondrial biogenesis, as well as the activities of the forkhead transcription factor (FOXO) family, which has been shown to modulate myogenesis [38,39]. Our data show that leucine increases SIRT-1 while SIRT-1 knockdown suppressess leucine-induced expression of mitochondrial regulatory genes, indicating that leucine-induced mitochondrial biogenesis is mediated, in part, by SIRT-1.

Mitochondria play a key role in the regulation of calcium ion homeostasis by serving as a buffer for cytosolic calcium [40]. In skeletal muscle fibers, the calcium buffering capacity of mitochondria is tightly linked to mitochondrial oxidative phosphorylation and may also be involved in associated gene expression [41]. Indeed, Ca^2+ ^signaling plays a role in modulating muscle cellular phenotypic adaptations via the Ca^2+^/calmodulin (CaM)-dependent phosphatase calcineurin (CnA) and Ca^2+^/CaM-dependent kinases, such as calcium/calmodulin dependent protein kinases (CaMK) I and II [34]; this effect regulates hypertrophic growth in response to overload to direct muscle fiber type switch gene expression and mitochondrial biogenesis. Although our data do not show a marked independent effect of nifedipine, it attenuated the effects of calcitriol on mitochondrial biogenesis and related gene expression, suggesting that calcium signaling plays a role in calcitriol regulation of mitochondrial biogenesis.

Energy partitioning between adipose tissue and skeletal muscle has been previously demonstrated [42-44]. Indeed, our previous data indicate that co-culture of muscle cells with adipocytes results in decreased fatty acid oxidation in muscle cells, and this effect is associated with modulation of cytokine expression and production. Consistent with this, co-culture with adipocytes or use of adipocyte-conditioned medium suppressed skeletal muscle mitochondrial abundance in the present study, indicating that mitochondrial biogenesis may mediate for leucine and calcitriol-induced regulation of fatty acid oxidation. Recent data demonstrate that tumor necrosis factor alpha (TNFα) down-regulates mitochondrial biogenesis in both white adipose tissue and muscle, while deletion of the TNF receptor in obese mice restores mitochondrial biogenesis; these effects maybe mediated by regulation of endothelial nitric oxide synthase (eNOS) production [45]. Adiponectin is also likely to play a role in the regulation of muscle mitochondrial biogenesis by adipocytes, as its expression is reduced with excess adiposity. Notably, adipocyte adiponectin secretion is regulated by SIRT-1 [46], although the role of this cytokine in mediating the cross-talk between adipocyte and muscle cells in regulating mitochondrial biogenesis is not yet clear.

We also found leucine regulation of mitochondrial mass in muscle cells and adipocytes to be associated with the stimulation of oxygen consumption. This observation provided further functional evidence for the modulation of mitochondrial biogenesis and energy metabolism by leucine. This effect is specific to leucine and is likely due to its role in stimulating protein synthesis and associated metabolic demand for energy [7], as another branched chain amino acid (valine) had no effect in this system.

We have utilized mitochondrial abundance, as measured by the fluorescent dye NAO, as an indicator of mitochondrial biogenesis. While these measurements cannot exclude the possibility that mitochondrial size, rather than number, was affected, the supporting data from both mitochondrial regulatory genes, such as PGC1α, which is well recognized to stimulate mitochondrial biogenesis, and mitochondrial component genes (e.g. cytochrome c oxidase) are indicative of an increase in mitochondrial number.

In summary, the present data demonstrate that leucine and calcitriol modulate energy metabolism, in part, through regulation of mitochondrial biogenesis, with leucine promoting fatty acid oxidation and mitochondrial biogenesis while calcitriol exerts the opposite effect. These data also support our previous observations of cross-talk between muscle cells and adipocytes in modulation of energy metabolism via secreted molecules from both cell types.

## Competing interests

The authors declare that they have no competing interests.

## Authors' contributions

XS and MBZ conceived of the study and jointly designed it and drafted the manuscript. XS conducted all laboratory analysis. Both authors have read and approve of the final manuscript.
